# Central nervous system tumors in patients coming from areas of conflict in the Middle East/North Africa region: an experience from King Hussein Cancer Center

**DOI:** 10.3389/fonc.2023.1087987

**Published:** 2023-05-17

**Authors:** Mouness Obeidat, Jamil Nazzal, Sarah Al Sharie, Ahmed Mahmoud Al-Azzam, Ahmad Maswadeh, Haneen Al-Abdallat, Layan Ismail, Marah Alkderat, Ro’ya Hzayen, Yasmeen Al-Sheble, Asem Mansour, Maysa Al-Hussaini

**Affiliations:** ^1^Department of Neurosurgery, King Hussein Cancer Center, Amman, Jordan; ^2^Office of Scientific Affairs and Research, King Hussein Cancer Center, Amman, Jordan; ^3^Faculty of Medicine, Yarmouk University, Irbid, Jordan; ^4^School of Medicine, The University of Jordan, Amman, Jordan; ^5^Department of Radiology, King Hussein Cancer Center, Amman, Jordan; ^6^Department of Pathology and Laboratory Medicine, King Hussein Cancer Center, Amman, Jordan

**Keywords:** cancer care facilities, oncology service, areas of conflict, MENA region, Arab countries, central nervous tumors, financial burden

## Abstract

**Introduction:**

The global cancer burden has been disproportionately shifting towards low- and middle-income countries (LMICs). Limited availability and accessibility to screening, treatment and surveillance, increase in the prevalence and lack of control of risk factors, and underdeveloped healthcare infrastructures have greatly contributed to the disparity in the global cancer burden.

**Methods:**

A retrospective cohort study was conducted that included adult and pediatric patients with an established diagnosis of Central Nervous System (CNS) tumors including brain or spinal tumors of which different demographic, clinical characteristics, and financial burden were presented.

**Results:**

749 patients were included stemming from various countries in the Middle East/North Africa (MENA) region including Libya (34.2%), Palestine (19.8%), Iraq (15.4%), Syria (14.6%) Yemen (14.5%), and Sudan (1.5%). Most patients were adults (66%) with a median age of 34-year-old. 104 patients had died (13.9%), 80 patients were still alive (10.7%) and most of the patients (n= 565, 75.5%) were lost to follow-up. The added cost of managing these patients is 10,172,935 Jordanian Dinars (JOD), with King Hussein Cancer Foundation (KHCF) covering around 34.3% of the total cost.

**Conclusion:**

Our study aimed at taking a closer look at patients coming from areas of conflict in the MENA region diagnosed and treated for CNS tumors at King Hussein Cancer Center (KHCC) over a 12-year period. It was found that even with the contributions of the Jordanian sources almost half of the patients were faced with the entire financial burden of treatment alone.

## 1 Introduction

The global cancer burden has been disproportionately shifting towards low- and middle-income countries (LMICs) with more than 50% of the 14.1 million cancer cases and with a projected 60% increase in the cancer burden by 2030 (1). Limited availability and accessibility to screening, treatment and surveillance, increase in the prevalence and lack of control of risk factors, and underdeveloped healthcare infrastructures have greatly contributed to the disparity in the global cancer burden (2). These problems have been further augmented by the disintegration of the healthcare systems due to the protracted conflicts prevalent in multiple countries in the Middle East and North Africa (MENA) region (3). A 2016 survey in Syria revealed a severe scarcity in cancer diagnosis and treatment resources including a shortage of specialized physicians, limited or lack of imaging modalities such as Magnetic Resonance Imaging (MRI), genetic testing, radiation therapy, bone marrow transplantation, and clinical trials (4). Similar conditions can be expected in multiple regions where long-lasting conflicts have been raging for decades. King Hussein Cancer Center (KHCC) is the largest comprehensive specialized cancer care institute in Jordan and one of the largest in the MENA region, and thus it has become a hub for many patients from neighboring countries seeking specialized cancer care, as well as many refugees currently housed in Jordan. Central nervous system (CNS) tumors are relatively rare tumors. However, they are still a significant cause of cancer related mortality and morbidity, specifically in children and young adults where they approximately cause 30% and 20% of cancer related mortalities, respectively (5). One of the most important prognostic factors in these tumors is the diagnostic interval which is defined as the time between symptom onset and the establishment of a diagnosis. Coven SL et al. found the median diagnostic interval of 42 days in a population of 146 American children (6), while Lu P et al. reported 97 days in a population of 433 Chinese children (7). Several publications addressed the overall burden of cancer among refugees. Mansour A et al. reviewed the burden of Syrian Refugees at KHCC including the cost burden. It was estimated that the cost of treating 869 Syrian patients with cancer was 15.6 million Jordanian Dinars (JOD) (22.1 million USD) annually; an important limiting factor to the access of proper and timely treatment (8). This has been reiterated in several other publications in countries from outside Jordan hosting Syrian refugees (9, 10). Mansour R et al. reviewed the burden of cancer among Palestinians living in the Palestinian Territories under the Israeli occupation that received treatment at KHCC in 2018 and 2019. Interestingly, CNS tumors were among the most common in which the initial diagnosis was changed among pathology reviewed at KHCC. However, detailed description of the discordant diagnosis was not mentioned in the paper (accepted for publication in Frontiers in Oncology). Most of the aforementioned literature discusses cancer in general, including description of the most common cancers (11). Literature on individual cancer types is scarce, but when available, would also focus on the most common types of cancer including breast in female patients (12). Focusing on pediatric cancer in children, a paper in 2020 comparing Syrian refugees to Turkish children with cancer reported significant differences in median age and median follow-up, metastatic or advanced-stage disease, relapse or progression and poor compliance to treatment and lower rates of overall and progression free survival in Syrian compared to Turkish patients (13). Interestingly, Syrian refugees were the focus of many studies, and other cancer patients from other areas of conflict were rarely reported, including patients from Yemen, Palestine, Libya, Iraq, and Sudan. Also, CNS tumors form only a small number of cases, thus when lumped with other tumors, the detailed description of the features unique to CNS tumors is barely presented.

We aim to describe CNS tumors among patients coming from countries in conflict in the MENA region, including the demographics, the diagnostic challenges and the financial burden and sources of funding in this group of patients managed at KHCC over a 12-year period.

## 2 Methods

### 2.1 Study design and participants

A retrospective cohort study was conducted that included adult and pediatric patients with an established diagnosis of CNS tumors including brain or spinal tumors (ICD-O Codes between C70.0-C72.9) that were referred to KHCC from countries/areas with ongoing conflicts, or patients residing in Jordan from any of the following countries; Iraq, Libya, Palestine, Sudan, Syria, and Yemen between 2010 and 2021.

The hospital electronic medical records, radiology reports and pathology reports were used to extract the following data including demographics, tumor primary site based on the International Classification of Diseases for Oncology, 3rd Edition (ICD-O-3), tumor type and grade as originally classified and then reclassified based on the 2021 WHO CNS classification, type of treatment including surgery, chemotherapy, and radiotherapy at both KHCC and other centers outside KHCC, and when available, survival status. The financial records were obtained from the Financial Department at KHCC, which included the cost of the management of the case, as well as the source of funding. This study was approved under number 22KHCC153.

### 2.2 Statistical analysis

Descriptive statistics were used for patient characteristics and clinical features of the study sample including describing demographics, histopathology, and sources of funding. Frequency with percentage was used to describe categorical variables.

Patients with non-applicable data in different variables, and patients who were lost to follow-up or who had a single visit to KHCC were excluded before conducting analyses. Pearson’s Chi-square test was used to compare differences between categorical variables in different nationalities.

All data analyses were conducted using Stata version 17 software (StataCorp. 2021. Stata: Release 17. Statistical Software. College Station, TX: StataCorp LLC.). The statistical significance was set at a 2-sided P<0.05.

## 3 Results

### 3.1 Demographics

As demonstrated in **Table 1**, this cohort included patients from areas of conflicts in the MENA region of which 256 patients were from Libya (34.2%), 148 from Palestine (19.8%), 115 from Iraq (15.4%), 110 from Syria (14.6%), 109 from Yemen (14.5%), and 11 from Sudan (1.5%). Of the total 749 patients, 429 were males (57.3%). The difference in gender among the included patients of the countries of conflict was statistically significant (p = 0.018). The median age at diagnosis was 34-years-old, 494 (66%) patients were adults and 255 (44%) were children or adolescents (< 18 years old).

**Table 1:** Demographics, clinical characteristics and outcome of included patients from areas of conflict.

	Total (n = 749)	Iraq (n = 115)	Libya (n = 256)	Palestine (n = 148)	Sudan (n = 11)	Syria (n = 110)	Yemen (n = 109)	*p-value*
**Age (pediatrics/adults)**								0.382
Adults (18 years and older)	494/749 (66%)	76/115 (66.1%)	181/256 (70.7%)	92/148 (62.2%)	7/11 (63.64%)	66/110 (60%)	72/109 (66.1%)	
Pediatrics (Younger than 18 years)	255/749 (34%)	39/115 (34.9%)	75/256 (29.3%)	56/148 (38.8%)	4/11 (37.36%)	44/110 (40%)	37/109 (33.9%)	
**Gender**								**0.018**
Female	320/749 (42.7%)	49/115 (42.6%)	123/256 (48%)	55/148 (37.2%)	8/11 (72.8%)	36/110 (32.7%)	49/109 (45%)	
Male	429/749 (57.3%)	66/115 (57.4%)	133/256 (52%)	93/148 (62.8%)	3/11 (27.2%)	74/110 (67.3%)	60/109 (55%)	
Surgery								
*At KHCC*								0.696
Yes	187/749 (25%)	23/40 (57.5%)	81/136 (59.6%)	34/67 (50.7%)	1/3 (33.3%)	28/46 (60.9%)	20/31 (64.5%)	
No	136/749 (18.1%)	17/40 (42.5%)	55/136 (40.4%)	33/67 (49.3%)	2/3 (66.7%)	18/46 (39.1%)	11/31 (35.5%)	
Not Applicable	426/749 (56.9%)	75/749 (10%)	120/749 (16%)	81/749 (10.8%)	8/749 (10.1%)	64/749 (8.5%)	78/749 (10.4%)	
*Outside of KHCC*								0.589
Yes	197/749 (26.3%)	28/28 (100%)	84/85 (98.8%)	34/34 (100%)	2/2 (100%)	21/22 (95.5%)	28/28 (100%)	
No	2/749 (0.3%)	0/28 (0%)	1/85 (0.2%)	0/34 (0%)	0/2 (0%)	1/22 (4.5%)	0/28 (0%)	
Not Applicable	550/749 (73.4%)	87/749 (11.6%)	171/749 (22.8%)	114/749 (15.2%)	9/749 (1.2%)	88/749 (11.7%)	81/749 (10.8%)	
Chemotherapy								
*At KHCC*								**0.03**
Yes	169/749 (22.6%)	20/115 (17.4%)	61/256 (23.8%)	45/147 (30.6%)	3/11 (27.3%)	25/110 (22.7%)	15/109 (13.8%)	
No	579/749 (77.3%)	95/115 (82.6%)	195/256 (76.2%)	102/147 (69.4%)	8/11 (72.7%)	85/110 (77.3%)	94/109 (86.2%)	
Not Applicable	1/749 (0.1%)	0/749 (0%)	0/749 (0%)	1/749 (0.1%)	0/749 (0%)	0/749 (0%)	0/749 (0%)	
*Outside of KHCC*								0.369
Yes	48/749 (6.4%)	4/43 (9.3%)	26/133 (19.5%)	9/59 (15.3%)	0/2 (0%)	5/40 (12.5%)	4/47 (8.5%)	
No	276/749 (36.9%)	39/43 (90.7)	107/133 (80.5%)	50/59 (84.7%)	2/2 (100%)	35/40 (87.5%)	43/47 (91.5%)	
Not Applicable	425/749 (56.7%)	72/749 (9.6%)	123/749 (16.4%)	89/749 (11.9%)	9/749 (1.2%)	70/749 (9.3%)	62	
Radiotherapy								
*At KHCC*								0.084
Yes	306/749 (40.9%)	51/115 (44.34%)	103/256 (40.2%)	72/148 (48.6%)	6/11 (54.5%)	37/110 (33.6%)	37/109 (33.9%)	
No	443/749 (59.1%)	64/115 (55.65%)	153/256 (59.8%)	76/148 (51.4%)	5/11 (45.4%)	73/110 (66.4)	72/109 (66.1%)	
Not Applicable	0	0	0	0	0	0	0	
*Outside of KHCC*								**0.043**
Yes	56/749 (7.4%)	3/40 (7.5)	33/123 (26.8%)	6/53 (11.3%)	0/2 (0%)	5/35 (14.3%)	9/47 (19.1%)	
No	244/749 (32.6%)	37/40 (92.5%)	90/123 (73.2%)	47/53 (88.7%)	2/2 (100%)	30/35 (85.7%)	38/47 (80.9%)	
Not Applicable	449/749 (60%)	75/749 (10%)	133/749 (17.8%)	95/749 (12.7%)	9/749 (1.2%)	75/749 (10%)	62/749 (8.3%)	
Discrepancy in Diagnosis								0.445
Major	26/749 (3.5%)	4/115 (3.5%)	13/256 (5.1%)	4/148 (2.7%)	0/11 (0%)	4/110 (3.6%)	1/109 (0.9%)	
Minor	4/749 (0.5%)	1/115 (0.8%)	1/256 (0.4%)	0/148 (0%)	0/11 (0%)	0/110 (0%)	2/109 (1.8%)	
No change in diagnosis	719/749 (96%)	110/115 (95.7%)	242/256 (94.5%)	144/148 (97.3%)	11/11 (100%)	106/110 (96.4%)	106/109 (97.3%)	
Outcome								0.027
Alive	80/749 (10.7%)	11/115 (9.6%)	14/256 (5.5%)	29/148 (19.6%)	0/11 (0%)	23/110 (20.9%)	3/109 (2.8%)	
Dead	104/749 (13.9%)	16/115 (13.9%)	38/256 (14.8%)	26/148 (17.6%)	2/11 (18.2%)	17/110 (15.5%)	5/109 (4.6%)	
Lost to follow-up	557/749 (74.4%)	85/115 (73.9%)	203/256 (79.3%)	93/148 (62.8%)	9/11 (81.8%)	68/110 (61.8%)	99/109 (90.8%)	
Single visit	8/749 (1.1%)	3/115 (2.6%)	1/256 (0.4%)	0/148 (0%)	0/11 (0%)	2/110 (1.8%)	2/109 (1.8%)	

Bolded values refer to statistically significant p-value (p < 0.05).

### 3.2 Clinical characteristics

The most common sites of CNS tumors in order were cerebrum including thalamus (n=363, 48.5%), meninges (n=130, 17.4%), cerebellum (n=126, 16.8%), brainstem (n=71, 9.5%), spinal cord (n=34, 4.5%), and cranial nerves (n=22, 2.9%) followed by other nervous systems sites (n=3, 0.4%). Based on the 2021 WHO Classification of Tumors of the Central Nervous System, the most common type of tumors encountered in this study were gliomas, glioneuronal tumors, and neuronal tumors (n=446, 59.5%) followed by meningiomas (n=131, 17.5%). However, the least encountered tumors were those of the sellar region, germ cell tumors, and choroid plexus tumors (0.3%, 0.5%, and 0.7% respectively). The majority of tumors were categorized as grade 4 WHO CNS tumors (n=341, 45.5%) followed by grade 1, 2, and 3 (n=233, 31.1%, n=89, 11.9%, and n=73, 9.7% respectively). **Table 2** shows further details of the various diagnostic categories.

**Table 2:** Primary CNS tumors sites, type, and grade based on the WHO CNS tumors classification.

	Total (n = 749)	Iraq (n = 115)	Libya (n = 256)	Palestine (n = 148)	Sudan (n = 11)	Syria (n = 110)	Yemen (n = 109)
Primary Sites							
Cerebrum including thalamus	363/749 (48.5%)	60/115 (52.2%)	120/255 (47.1%)	78/147 (53.1%)	7/11 (63.6%)	54/110 (49.1%)	44/108 (40.7%)
Meninges	130/749 (17.4%)	19/115 (16.5%)	56/255 (22%)	15/147 (10.2%)	1/11 (9.1%)	14/110 (12.7%)	25/108 (23.1%)
Cerebellum	126/749 (16.8%)	21/115 (18.3%)	32/255 (12.5%)	29/147 (19.7%)	2/11 (18.2%)	19/110 (17.3%)	23/108 (21.3%)
Brainstem	71/749 (9.5%)	10/115 (8.7%)	25/255 (9.8%)	10/147 (6.8%)	1/11 (9.1%)	13/110 (11.8%)	12/108 (11.1%)
Spinal Cord	34/749 (4.5%)	4/115 (3.5%)	11/255 (4.3%)	11/147 (7.5%)	0/11 (0%)	6/110 (5.5%)	2/108 (1.9%)
Cranial and paraspinal nerves	22/749 (2.9%)	1/115 (0.9%)	11/255 (4.3%)	4/147 (2.7%)	0/11 (0%)	4/110 (3.6%)	2/108 (1.9%)
Other nervous system sites	3/749 (0.4%)	0/115 (0%)	1/256 (0.4%)	1/148 (0.7%)	0/11 (0%)	0/110 (0%)	1/109 (0.9%)
Tumor Type/2021 CNS Tumors Classification							
Gliomas, glioneuronal and neuronal tumors	446/749 (59.5%)	77/115 (66.9%)	143/255 (56.1%)	89/146 (60.9%)	9/11 (81.8%)	69/109 (63.3%)	59/109 (54.1%)
Meningioma	131/749 (17.5%)	20/115 (17.4%)	56/255 (22%)	15/146 (10.2%)	1/11 (9.1%)	14/109 (12.8%)	25/109 (23%)
Embryonal tumors	97/749 (13%)	11/115 (9.6%)	24/255 (9.4%)	26/146 (17.8%)	1/11 (9.1%)	18/109 (16.5%)	17/109 (15.6%)
Cranial and paraspinal nerve tumors	31/749 (4.1%)	2/115 (1.7%)	16/255 (6.3%)	8/146 (5.5%)	0/11 (0%)	2/109 (1.8%)	3/109 (2.75%)
Hematolymphoid tumors	17/749 (2.3%)	4/115 (3.5%)	5/255 (2%)	3/146 (2.1%)	0/11 (0%)	3/109 (2.8%)	2/109 (1.8%)
Mesenchymal, non-meningothelial tumors	11/749 (1.5%)	0/115 (0%)	7/255 (2.7%)	2/146 (1.4%)	0/11 (0%)	0/109 (0%)	2/109 (1.8%)
Choroid plexus tumors	5/749 (0.7%)	1/115 (0.9%)	1/255 (0.4%)	1/146 (0.7%)	0/11 (0%)	1/109 (0.9%)	1/109 (0.9%)
Germ cell tumors	4/749 (0.5%)	0/115 (0%)	1/255 (0.4%)	1/146 (0.7%)	0/11 (0%)	2/109 (1.8%)	0/109 (0%)
Tumors of the sellar region	2/749 (0.3%)	0/115 (0%)	2/255 (0.7%)	0/146 (0%)	0/11 (0%)	0/109 (0%)	0/109 (0%)
Melanocytic tumors	1/749 (0.1%)	0/115 (0%)	0/255 (0%)	1/146 (0.7%)	0/11 (0%)	0/109 (0%)	0/109 (0%)
Not applicable	4/749 (0.5%)	0/115 (0%)	1/256 (0.4%)	2/148 (1.4%)	0/11 (0%)	1/110 (0.9%)	0/109 (0%)
WHO grade							
1	233/749 (31.1%)	34/114 (29.8%)	99/254 (39%)	33/145 (22.7%)	3/10 (0.3%)	27/105 (25.7%)	37/108 (34.3%)
2	89/749 (11.9%)	15/114 (13.2%)	25/254 (9.8%)	19/145 (13.1%)	0/10 (0%)	12/105 (11.5%)	18/108 (16.7%)
3	73/749 (9.7%)	13/114 (11.4%)	23/254 (9.1%)	21/145 (14.5%)	1/10 (0.1%)	6/105 (5.7%)	9/108 (8.3%)
4	341/749 (45.5%)	52/114 (45.6%)	107/254 (42.1%)	72/145 (49.7%)	6/10 (0.6%)	60/105 (57.1%)	44/108 (40.7%)
Not applicable	13/749 (1.8%)	1/115 (0.9%)	2/256 (0.8%)	3/148 (2%)	1/11 (9.1%)	5/110 (4.5%)	1/109 (0.9%)

Out of the 749 patients at KHCC, 187 patients underwent surgery (25%), 169 received chemotherapy (22.6%), and 306 received radiotherapy (40.9%). This is compared to 197 patients who had surgery (26.3%), 48 received chemotherapy (6.4%), and 56 received radiotherapy (7.4%) outside KHCC before receiving treatment at KHCC. There was a statistically significant difference in the number of patients between countries who did and did not receive chemotherapy at KHCC (*p* = 0.03) and radiotherapy outside of KHCC (*p* = 0.043).

Importantly, 26 (3.5%) patients had a major discrepancy in diagnosis when admitted to KHCC, 4 (0.5%) patients had minor changes in diagnosis and the rest of patients did not have any change in diagnosis (96%).

A hundred and four patients of the study population had died (13.9%) and 80 patients were still alive (10.7%) at time of collection of the data. Unfortunately, most of the patients (n= 565, 75.5%) were lost to follow-up, including 8 (1.1%) patients who had only a single visit to our center. Statistically, after excluding patients who were lost to follow-up and of single visits, there was a significant difference in the outcome between the countries in relation to survival (p = 0.027).

### 3.3 Sources of funding

The total cost of managing 749 patients was 10,172,935 JOD (equivalent to 14,348,467 USD). Patients receiving treatment at KHCC were funded either by Jordanian sources (n=171, 22.8%), by the corresponding government of the patient (n=242, 32.3%), by private insurance (n=14, 1.9%) or were self-funded (n=321, 42.9%). The amount of funds by each source in the included countries is demonstrated in **Table 3**. It is worth mentioning that Jordanian sources contributed 4,604,632 JOD (6,494,626.2 USD), accounting for 45.3% of the total cost of treatment for the patients, out of which King Hussein Cancer Foundation (KHCF) covered the costs of 3,492,383 JOD (4,925,849 USD), accounting for 34.3% of the total cost.

**Table 3:** Funding sources and amounts for patients with CNS tumors coming from areas of conflict receiving treatment at KHCC.

Number of patients	Total (n=749)	Iraq (n=115)	Libya (n=256)	Palestine (n=148)	Sudan (n=11)	Syria (n=110)	Yemen (n=109)	*p*-value
Source of Funding (no of patients,%) Cost (JOD) (% from the overall cost)								
**Jordan**	171 (22.8%) 4,604,632 JOD (45.3%)	23 (20%) 632,228 JOD (58%)	7 (2.7%) 102,535 JOD (3.2%)	68 (46%) 2,240,698 JOD (66.6%)	1 (9.1%) 3,673 JOD (5.6%)	59 (53.6%) 1,466,644 JOD (83.8%)	13 (11.9%) 158,854 JOD (22.1%)	**0.000**
Jordanian government	34 (4.5%) 1,050,638 JOD (10.3%)	0 (0%) 0 JOD (0%)	1 (0.4%) 6,300 JOD (0.2%)	27 (18.2%) 849,308 JOD (25.2%)	0 (0%) 0 JOD (0%)	4 (3.6%) 189,842 JOD (10.9%)	2 (1.8%) 5,188 JOD (0.7%)	
KHCF	124 (16.6%) 3,492,383 JOD (34.3%)	22 (19.1%) 627,795 JOD (57.6%)	1 (0.4%) 44,436 JOD (1.4%)	40 (27%) 1,391,305 JOD (41.3%)	1 (9.1%) 3,673 JOD (5.6%)	50 (45.5%) 1,273,778 JOD (72.8%)	10 (9.2%) 151,396 JOD (21.0%)	
Private sector	13 (1.7%) 61,611 JOD (0.6%)	1 (0.9%) 4,433 JOD (0.4%)	5 (2%) 51,799 JOD (1.6%)	1 (7%) 85 JOD (0.03%)	0 (0%) 0 JOD (0%)	5 (4.6%) 3,024 JOD (0.2%)	1 (0.9%) 2,270 JOD (0.3%)	
**Corresponding government**	242 (32.3%) 3868289 JOD (38%)	0 (0%) 0 JOD (0%)	199 (77.7%) 2,873,772 JOD (90.4%)	43 (29%) 994,517 JOD (29.5%)	0 (0%) 0 JOD (0%)	0 (0%) 0 JOD (0%)	0 (0%) 0 JOD (0%)	**0.000**
**Private insurance**	14 (1.9%) 132,848 JOD (1.3%)	1 (0.9%) 17,860 JOD (1.6%)	6 (2.3%) 32,396 JOD (1%)	0 (0%) 0 JOD (0%)	0 (0%) 0 JOD (0%)	0 (0%) 0 JOD (0%)	7 (6.4%) 82,592 JOD (11.5%)	**0.003**
**Self-funded**	321 (42.9%) 1,567,166 JOD (15.4%)	91 (79.1%) 440,772 JOD (40.4%)	43 (16.8%) 171,142 JOD (5.4%)	37 (25%) 131,528 JOD (3.9%)	10 (90.9%) 62,392 JOD (94.4%)	51 (46.4%) 282,623 JOD (16.2%)	89 (81.7%) 478,709 JOD (66.5%)	**0.000**
**Total amount paid**	10,172,935 JOD	1,090,860 JOD	3,179,845 JOD	3,366,743 JOD	66,065 JOD	1,749,267 JOD	720,155 JOD	

Bolded values refer to statistically significant p-value (p < 0.05).

The countries of which individuals received the most of Jordanian sources of funding were Palestine (n=68, 2,240,698 JOD/3,158,579 USD) and Syria (n=59, 1,466,644 JOD/2,067,441 USD). In addition to that, the number of individuals receiving funding from their corresponding government was the highest in patients of Libyan nationality (n=199, 2,873,772 JOD/4,051,038 USD). Moreover, Yemenis patients receiving funding from private insurance were the highest among other countries (n=7, 82,592 JOD/116,426 USD) followed by Libyan patients (n=6, 32,396 JOD/45,667 USD). Furthermore, the countries of which individuals self-funded their medical expenses the most were Iraq (n=91, 440,772 JOD/621,338 USD) and Yemen (n=89, 478,709 JOD/674,816 USD). It is worth mentioning that the difference between the number of patients of each country in regard to different funding sources was statistically significant (*p* < 0.05).

## 4 Discussion

This study aimed to describe patients with an established diagnosis of a CNS tumor who came from areas of ongoing conflicts in the MENA region to receive treatment at KHCC in Jordan. To our knowledge, our study is the first to take a closer look at this patient population as the literature is scarce. We aimed to describe the challenges they faced in attaining proper healthcare including both diagnostic workup and appropriate treatment as well as the financial burden that they as well as the Jordanian sources and the KHCF faced in order to achieve it.

Our study included a total of 749 patients stemming from various countries in the MENA region including 256 patients were from Libya (34.2%), 148 from Palestine (19.8%), 115 from Iraq (15.4%), 110 from Syria (14.6%), 109 from Yemen (14.5%), and 11 from Sudan (1.5%). Sixty-six percent of the patients were adults and the median age of diagnosis was 34 years old with a statistically significant male predominance (57.3%). The majority of patients had grade 4 tumors at presentation (45.5%) according to the WHO CNS tumor classification, with gliomas, glioneuronal, and neuronal tumors (59.5%) being the most common, followed by meningiomas (17%). The majority of the tumors were found in the cerebrum (48.5%) followed by the meninges (17.4%) and the cerebellum (16.8%). The treatment regimens at KHCC mostly consisted of radiotherapy (40.9%), surgery (25%), and chemotherapy (22.6%). Interestingly, 26 (3.5%) patients had a major discrepancy in diagnosis when admitted to KHCC while the majority had their diagnosis confirmed (96%) and received treatment. As of the writing of this study, 10.7% of the patients were confirmed to be alive and 13.9% were deceased, while the rest of the patients (75.5%) were lost to follow-up. Among the accounted-for patients the overall survival was significantly different between different nations (p = 0.027).

The countries of which individuals were recorded to receive the majority of the total fund were Palestine (3,366,743 JOD/4,745,959 USD) and Libya (3,179,845 JOD/4,482,497 USD). This might be explained by the fact that Libyan (n=61) and Palestinian (n=45) patients were the highest individuals to receive chemotherapy regiments at KHCC compared to patients of other countries. Moreover, the discrepancy of the number of patients receiving chemotherapy may be attributed to the prolonged duration needed to complete regimens of chemotherapy leading them to prefer receiving their treatment in their home countries. Additionally, these prolonged durations may require higher expenses on the patients, their funding sources, or on their companions who may not be covered by funding policies.

The country in which patients received the least amount of funding was Sudan (66,065 JOD/93,129 USD). Of note, Sudan recorded the highest percentage of deceased patients (18.2%). On the contrary, Yemen was the second least funded country (720,155 JOD/1,015,172 USD) with the least percentage of deceased patients (4.6%). It is important to note that the aforementioned countries recorded the highest number of individuals who were lost to follow up. Based on the previous observation we could not correlate the overall survival with the amount of received fund for each country.

In a study by Fan et al. investigating the burden and trends of brain and central nervous system cancers in 2019, the percentage of deaths in high-middle income countries was 70.33% and 82.82% in LMICs (14). After excluding patients who were lost to follow-up, we have found that 56.52% of patients coming from areas of conflict in the MENA region were dead.

CNS tumors affect both children and adults and represent a substantial source of morbidity and mortality worldwide (15), especially in children and young adults where they approximately account for 30% and 20% of all cancer-related deaths respectively (5). The most common histological subtype of primary CNS cancer are gliomas; a large group of malignant brain tumors ranging from high-grade glioma (glioblastoma) to low-grade gliomas (astrocytoma, oligodendroglioma) (16). Glioblastomas, the most common primary brain cancer of glial origin, are almost always fatal within 2 years of diagnosis even with extensive surgical and medical therapy (17). In children, common histologies include medulloblastoma, germ-cell tumors, astrocytoma, brainstem gliomas, and ependymomas. Although these tumors are associated with high rates of morbidity and mortality, long-term survival can be achieved with comprehensive treatment strategies (18). The global burden of CNS cancer seems to be increasing significantly. A recent study found that in 2016 the global incidence of CNS tumors was approximately 330,000 cases with an estimated 227,000 deaths attributed to CNS tumors that same year. They also found that in the period between 1990 and 2016, the age-standardized incidence rates of CNS cancer increased globally by 17.3% (19). The effects that CNS tumors pose are underestimated by the incidence rates due to their high mortality rates and inherently disabling effects often rendering patients unfunctional for the rest of their lives (20). The burden of CNS tumors on healthcare systems is great and is often augmented by the fact that effective treatment is multimodal requiring access to an array of healthcare services including but not limited to chemotherapy, radiotherapy, and neurosurgical care (21). This highly specialized care is not widely available, especially in poor countries where war and conflict have been raging for decades forcing patients and their families to go through many difficulties just to gain access only to be faced with a lengthy and often pricy treatment plan putting further strain on their fragile livelihoods.

The total financial cost for the treatment of the 749 patients was about 10,172,935 JOD (equivalent to 14,348,467 USD). The main sources of funding included Jordanian sources, the corresponding government of the patients, private insurance, and self-funding by the patients or their families. Most of the patients’ corresponding governments had no contribution to the treatment costs with the only exception being the Libyan and Palestinian governments contributing 90.4% and 29.5% of the total treatment costs respectively. About 42.9% (n=321) of the patients relied solely on out-of-pocket expenditure with only 1.9% (n=14) having any form of private insurance. The average out-of-pocket cost per patient was approximately 4882 JOD (6885 USD), which would constitute a massive financial burden given that the majority of patients come from low-income countries (Gross national income per capita of 1,085 USD or less, n=230), LMICs (Gross national income per capita of 1,086-4,255 USD, n=148) and upper-middle income countries (Gross national income per capita of 4,256-13,205 USD, n=371) (22), further emphasizing the financial hardships faced by these patients while dealing with the physical and psychological hardships of the disease itself. Even while limited by scarce resources, the Jordanian sources contributed about 45.3% (6,494,626.2 USD) of the total treatment costs, out of which the KHCF accounted for 34.3% of the total treatment cost.

The histopathological diagnosis was entirely changed for 3.5% (n=26) of the patients and showed minimal discrepancies in 0.5% (n=4) of the patients, highlighting the need for specialized neuropathologists not only during the treatment stage but also before coming to a conclusive diagnosis. Even though the majority of patients did not have any discrepancies in the diagnosis, a multitude of workups were done to either confirm or establish the diagnosis. Many of the patients included in the study did not have access to proper cancer diagnosis and treatment resources before presenting at KHCC, including specialized physicians, imaging modalities, and multiple blood tests that would be necessary for diagnosis and formulation of a treatment plan.

The biggest limitation of our study was the fact that the majority of the patients were lost to follow-up (75.5% n=565), a problem that commonly happens following the completion of treatment in many cancer patients. In a large cancer center in the United States, 22% (858 out of 3924) of breast cancer survivors were lost to follow-up after 10 years (23). In another study in the United Kingdom, 46%(256 out of 550) of cervical cancer patients were lost to follow-up after 3 years (24). However, this problem seems to be more prominent in developing countries as a study done in India found that almost 80% of their 690 cervical cancer patients were lost to follow-up within a 3-year period (25). The majority of our patient population was not indigenous to the country of Jordan and so most of them returned to their countries following treatment making follow-up extremely costly and difficult. Perhaps with the rise of telemedicine, these patients may be able to continue to receive expert care from the comfort of their homes, although this might also be limited by the inability to perform necessary tests and investigations in their home country that lacks such resources, thus limiting the ability to monitor for long-term effects of their treatment (chemotherapy, immunotherapy, or radiotherapy), and the potential risks of developing second, cancers.

## 5 Conclusion

In conclusion, our study aimed at taking a closer look at patients coming from areas of conflict in the MENA region diagnosed and treated for CNS tumors at King Hussain Cancer Center. We described the demographics, the diagnostic challenges, and the financial burden and sources of funding in this group of patients managed at KHCC over a 12-year period. A small percentage of patients had major discrepancies in their diagnosis and the majority of patients were lost to follow-up, we also found that even with the contributions of the Jordanian sources almost half of the patients were faced with the entire financial burden of treatment alone.

## Data availability statement

The original contributions presented in the study are included in the article/supplementary material. Further inquiries can be directed to the corresponding author.

## Ethics statement

The studies involving human participants were reviewed and approved by King Hussein Cancer Center ethics committee. This study was waived from obtaining informed consent, as there was no direct interaction with participants or their legal guardian/ next of kin.

## Author contributions

Conceptualization: MO and MA-H. Methodology: SAS. Formal analysis: SAS and MA-H. Data Collection: AA-A, AhM, HA-A, LI, MA, RH, and YA-S. Writing – original draft: JN and SAS. Writing – review and editing: AsM and MA-H. Supervision: MO, MA-H, and AsM. All authors contributed to the article and approved the submitted version.
